# Abdominal Surgery in North Germany1Read before the San Francisco Medico-Chirurgical Society.

**Published:** 1894-06

**Authors:** Clinton Cushing

**Affiliations:** Professor of Gynecology, Cooper Medical College, San Francisco, Cal.; 636 Sutter Street


### ABDOMINAL SURGERY IN NORTH GERMANY.[1]

By CLINTON CUSHING, M. D.

Professor of Gynecology, Cooper Medical College, San Francisco, Cal.

In introducing the following remarks, upon what I saw of abdominal surgery in Dresden and Berlin, during the short time spent in those cities the past summer, I desire to pay a tribute to the courtesy of the gentlemen with whom I came in contact. I saw nothing but the greatest kindness shown to all who applied for privileges and opportunities to see and to investigate in professional matters. Socially, they evinced the most charming hospitality, and among the agreeable memories of the journey are the pictures of pleasant home life of the doctors in their own houses, the predominating features of which were simplicity, naturalness and sincerity.

The Royal Clinic for women, in Berlin, is presided over by Professor Olshausen. It is a substantial brick and stone structure, with all the modern improvements in the shape of electric lights and the best forms of sewerage and ventilation, baths, tiled floors and appliances for cleanliness. Special rooms are set apart for out-patients and for the preparation of patients who are to be confined. The Berlin Gynecological Society holds its meetings in the building.

A fine amphitheater has also recently been completed, where lectures and demonstrations are given by the professor and his assistants. It is scarcely necessary for me to tell you that the most scrupulous attention is paid to every detail, so as to insure cleanliness of places, persons and things. Indeed, I have never

---

1. Read before the San Francisco Medico-Chirurgical Society.

seen as much attention paid to the details, both before and during an operation, as in North Germany.

Operations are generally done early in the morning, six or seven o'clock, and usually the operation begins at the exact moment that had previously been designated. The operator and his assistants are dressed in clean linen garments, with long white gowns, and when the operation begins the door is locked and perfect silence reigns, broken only by the orders of the chief to his subordinates. No story-telling, laughing or anything else is permitted that might distract the attention of anyone from his duties. From what I could learn, ether is becoming more used in Germany than formerly, except where diseases of the kidneys exist.

Prof. Olshausen is a slender man of medium height, whom I should judge to be about fifty years of age; his black hair is getting a trifle thin on top, and a little gray at the temples. His eyes are bright and quick, and his expression very agreeable. His manner, that of a man whose time is all occupied. He has written one of the best books that we have on diseases of the ovaries. As an operator, he is very careful and painstaking, and he evidently believes that what is worth doing, is worth doing well.

One of the most noticeable features connected with his abdominal surgery is the almost universal use of catgut for ligatures and sutures. In the vaginal extirpation of the uterus and in the ligation of the pedicle after ovariotomy, only catgut ligatures are used. In closing the abdominal wall, three rows of running catgut sutures are made. The first one includes the peritoneum, the second the muscular and fibrous tissues, and the last the skin. In very fat women three or four retention stitches of silk are passed through all the structures of the abdominal wall, as a matter of security. The silk sutures are removed in a week, the catgut is not disturbed and is absorbed in about ten days. Prof. Olshausen showed me numerous cases where the union seemed to be all that could be desired.

The dressing for the abdominal wound is dry benzoate of soda and a layer of absorbent cotton, covered by a bandage. Drainage of the peritoneal cavity is not common in Berlin. Great pains is taken to control all hemorrhage and to cleanse the cavity as well as possible, and then to trust largely to the absorptive qualities of the peritoneum and the powers of the individual to resist any evil tendencies. Personally I do not agree with those who decry drainage, and am more and more convinced each year of its great value in many cases, especially where the peritoneal cavity has become infected with pus, and where there is much oozing of blood and serum.

The opening in the abdominal wall is made decidedly larger than with us, or by the English operators. This gives a larger field to work in, but it increases the shock and causes greater exposure of the abdominal cavity to cold, in my opinion increasing the risk, especially where the operation is a prolonged one. The Trendelenberg position I saw used but once or twice, and in spite of its manifest advantages in certain cases, operators have found that ordinarily the prone position is the best. I had come to this conclusion two years ago, after having used the Trendelenberg position in several cases, being convinced that the gravitation of blood and fluids into the upper part of the peritoneal cavity was a serious disadvantage.

In the removal of large uterine fibroids by abdominal section, there is a growing disposition to remove the entire uterus, including the cervix, and thus far with very good results. My observation leads me to the conclusion that success by this method rests almost entirely upon the fact of the free drainage below and the prevention of the infection of the site of operation from the vagina. With previous thorough cleansing of the vagina and the removal of the cervix, with its many diseased glands, and the subsequent filling of the vagina with antiseptic tampons, the peritoneal cavity is protected. Whether total removal will be followed by subsequent prolapse of the intestines into the vagina, can be determined only by trial.

If peritonitis develop after an abdominal section, no antiseptic is given. Sufficient opium is used to control pain, and a saline laxative is given every hour, assisted by large rectal enemas, until purgation ensues. Personally, I have found calomel, in two-grain doses every hour, the most practicable and useful remedy in these cases. Reopening the abdomen and washing and draining after the peritonitis has fully developed, has been followed by good results in a few cases; it is usually of little avail.

Dr. August Martin is one of the most prominent figures in gynecology in Berlin, by reason of the large amount of work that he does, and on account of his many writings. His father was the celebrated professor of obstetrics in the University of Berlin forty years ago, and was present at the accouchement of the Princess

Victoria, when the present Emperor was born. Dr. Martin is a very large man, weighing, I should judge, nearly 250 pounds, and is endowed with untiring energy and industry. He has a large private hospital and clinic, and I have seen him do five abdominal sections in one morning. He has private classes attendant upon his operations at certain seasons, and has a large following of students from all parts of the world. He is a typical German in appearance, is very busy, and has little time for anything outside of his professional duties. He is evidently working much too hard, and will break down unless he is more considerate for himself.

His methods in operating are different from any other that I have seen. In doing an abdominal section, he sits on a stool at the end of a low, iron table, with the patient's legs sticking out on each side over the end of the table and unsupported. He opens the abdomen rapidly, and, owing to his position, is enabled to use either hand with equal facility, in exploring the abdominal cavity and in operative manipulation. His assistants stand on each side of the patient, and are so expert that the operator seldom is compelled to ask for any instrument or appliance, for the proper thing is placed in his hand immediately it is wanted. His matron, Frau Horn, a short, thick-set, jolly, gray-haired, motherly-looking woman, always assists him in his work. She is very intelligent, and is the inventor of a number of instruments and appliances in use at the hospital.

As in Olshausen's clinic, catgut is largely used. In extirpating the uterus, *per vaginam*, no compression forceps are used; the broad ligaments are tied in sections with catgut. In laparatomy, he makes a large incision, and, with large, flat sponges, his assistants hold the intestines out of the way. Frau Horn made a suggestion that, in order, to prevent adhesions between the opposing surfaces of peritoneum after operations, a sponge saturated with olive oil, sterilized by boiling, should be introduced into the abdominal cavity, and the peritoneal surfaces given a coating of oil. This has been repeatedly done during the past few years, and apparently with good effects, but the final conclusion as to the value of the method has not yet been arrived at.

Martin's method of dealing with sub-peritoneal fibroids of the uterus is well known. The peritoneum over the tumor is laid open and the mass shelled out with the fingers and the handle of the scalpel, like a pea out of its pod, the sub-peritoneal tissues with the line of incision in the peritoneum being carefully closed with catgut and a running suture, a satisfactory method of dealing with sessile growths.

Martin is an ardent advocate of vaginal extirpation of the uterus for carcinoma. He has operated over 100 times, and in the last English edition of his book, translated by Dr. E. W. Cushing, of Boston, a most excellent work, he claims that in forty-four cases where it could be shown that the disease did not extend into the tissues outside the uterus, 70 per cent. remained well two years and a half after the operation.

The work of Leopold, of Dresden, is deserving of especial mention. His excellent hospital for women is under the patronage and control of the royal family of Saxony, and was formerly supervised by Winckel, now of Munich, who did much to make it famous. He was succeeded by Leopold, whose record in Cesarean section is remarkable. His last statistics that have come to my notice show forty-one Cesarean sections, with the loss of three mothers and the saving of a large proportion of the children. The frequency of such cases in Dresden is due to the large number of dwarfed and deformed people in Saxony, on account of poor food and overwork. Many of the poor women are brought in carts, long distances and in a deplorable state, and it is wonderful that so large a proportion recover.

Upon entering the hospital, the patient is taken into a large room, all clothing is removed, and a number of nurses set to work upon her, who scrub her with soap and hot water, inside and out. This is followed by antiseptic washes and douches *ad libitum.* The patient is then taken to the operating room, where antiseptic precautions of every kind are observed. The abdomen and uterus are rapidly opened, the child extracted and given to an assistant, the placenta removed and the incision in the uterus closed by deep interstitial silk sutures, unless there is reason to believe that the uterine cavity has already become septic. In this event, a Porro operation is done, the body of the uterus is removed and the stump treated externally, which was the case in the instance where I saw the operation last July.

I also saw Leopold operate in a case of extrauterine pregnancy of two months' standing. His methods are admirable, decisive, clean, rapid, and, what is most important, he is remarkably successful in what he undertakes. He is very cordial to visitors, and is surrounded by a large class of young graduates in medicine, many of whom reside in the hospital where the material for obstetrical and gynecological work is abundant.

Compared with other parts of the world, the surgery of North Germany gives the impression of being very satisfactory, the most pronounced features being scrupulous cleanliness and the administration of few drugs, the power of Nature and the absence of septic infection being, in the main, relied upon for recovery of the patient.

For the younger men in the profession, it is difficult to appreciate the remarkable advances that have been made in surgery during the past twenty-five years, especially since the era of Lister—an era that will stand out in all future histories of medicine as one of the most brilliant and remarkable, to be compared only with the great era of cellular pathology, and with the introduction into our art of instruments of precision, such as the clinical thermometer and the modern microscope.

The magnificent structure of modern surgery, that is being erected at the present day by active workers in all parts of the field, is likely to be enduring, from the fact that it is based, not so much upon individual opinion and skill, as was so markedly the case in former years, but upon the use of these same instruments of precision which leave no doubt as to the exact facts. There is now no chance for an argument between educated men as to whether a given specimen of sputum contains the bacillus of tuberculosis. The question of the exact temperature of the body in a given case of disease is at once determined accurately, and much speculation prevented and time saved.

We are, indeed, living in a golden age of progress and discovery; and the end is not yet, but great things are promised in the near future to those who are investigating the causes of disease. One of the most hopeful signs of the times is the simplification of methods and of ways and means of managing the sick, especially in surgical matters. In other words, mathematical exactness is slowly but surely taking the place of theory and guess-work.

To whom do we owe, in large part, these modern improvements in exact methods and good results? Undoubtedly to the plodding, painstaking, patient work of the Germans. It does not seem possible that any one can go to their schools of pathology without being filled with admiration for their labor and fidelity in working out problems to a successful issue, that we may the better understand the nature of disease.—*Occidental Medical Times.*

636 SUTTER STREET.

